# Prevalence of Helicobacter pylori infection among asymptomatic children in southeastern Brazil: a cross-sectional study

**DOI:** 10.1590/1516-3180.2021.0721.R2.03032022

**Published:** 2022-08-29

**Authors:** Ana Beatriz Marques Carlos, Vladimir Eliodoro Costa, Renata Kobayasi, Maria Aparecida Marchesan Rodrigues

**Affiliations:** I MSc. Postgraduate Student, Department of Pathology, Faculdade de Medicina de Botucatu (FMB), Universidade Estadual Paulista (UNESP), Botucatu (SP), Brazil.; II PhD. Coordinator, Stable Isotope Center, Instituto de Biociências de Botucatu (IBB), Universidade Estadual Paulista (UNESP), Botucatu (SP), Brazil.; III MD, PhD. Assistant Physician, Sírio Libanês Hospital, Department of Internal Medicine, Faculdade de Medicina da Universidade de São Paulo (FMUSP), Brazil.; IV MD, PhD. Full Professor, Department of Pathology, Faculdade de Medicina de Botucatu (FMB), Universidade Estadual Paulista (UNESP), Botucatu (SP), Brazil.

**Keywords:** Helicobacter pylori, Child, Prevalence, Children, Prevalence of *H. pylori* infection, Urea breath test

## Abstract

**BACKGROUND:**

The prevalence of *Helico bacter pylori* (*H. pylori*) infection is decreasing worldwide, but is still high in developing countries. We previously observed an *H. pylori* infection rate of 52% among children and adolescents with chronic non-ulcer dyspepsia.

**OBJECTIVE:**

To investigate the prevalence of *H. pylori* infection among asymptomatic children living in a single region and to evaluate the risk factors for this infection.

**DESIGN AND SETTING:**

Cross-sectional study in which 161 children aged 5-13 years (mean age 7.8 years), at a public school in Botucatu, state of São Paulo, southeastern Brazil, were assessed.

**METHOD:**

The children's *H. pylori* infection status was determined through the urea breath test and the risk factors for acquisition of the infection were determined based on a sociodemographic questionnaire.

**RESULTS:**

The overall prevalence of *H. pylori* infection was 20.5%: 18.7% among females and 22.2% among males. The results from the sociodemographic survey did not differ between children with and without *H. pylori* infection*.* 30.9% of the children had previous records of upper gastrointestinal symptoms, which consisted of *H. pylori* infection in only 26.5% of these cases. Family histories of gastritis and peptic ulcer disease were found in relation to 50% and 32.3% of the children with *H. pylori* infection respectively.

**CONCLUSION:**

The prevalence of *H. pylori* infection among asymptomatic children in southeastern Brazil is lower than that recorded among symptomatic children in the same region and similar to the prevalence of *H. pylori* infection observed in developed countries.

## INTRODUCTION

The prevalence of *Helicobacter pylori* (*H. pylori*) infection is decreasing worldwide, but is still high in developing countries.^1–3^ In Brazil, epidemiological studies have shown that the rates of *H. pylori* infection vary widely across different regions; for example, the highest rates have been reported in northern and northeastern regions (up to 90%), in contrast to lower prevalence in the southeastern region (less than 36%).^4,5^

We previously observed an *H. pylori* infection rate of 52% among children and adolescents with chronic non-ulcer dyspepsia in southeastern Brazil.^6,7^ This prompted us to investigate the prevalence of *H. pylori* infection among asymptomatic children living in a single community.

## OBJECTIVE

To investigate the prevalence of *H. pylori* infection among asymptomatic children using the urea breath test. Sociodemographic data and previous records of gastrointestinal symptoms were assessed to investigate risk factors for this infection.

## METHODS

This cross-sectional prospective study was conducted among children at a public school in the city of Botucatu city, São Paulo, southeastern Brazil, from October 2019 to February 2020. They were aged between 5 and 13 years and were living in the same geographical area as those of a previous study on symptomatic children^6,7^ The exclusion criteria were occurrences of therapy with antibiotics or antisecretory drugs within the preceding four weeks. A sociodemographic questionnaire was administered to the parents. Questions asking about previous records of upper gastrointestinal symptoms in the last year, like nausea, vomiting, epigastric pain, post-prandial fullness and nocturnal pain, were included in the questionnaire.

*H. pylori* infection was evaluated through the urea breath test. This was applied to the children in the school, in the morning, before the recess, when the children had had a fasting time of approximately two hours. A baseline breath sample was collected, and then the children received a solution containing ^13^C-labeled urea (^13^C-urea): 50 mg for children ≤ 30 kg and 75 mg for children > 30 kg, dissolved in 80 ml of water. After 15 minutes, the final breath sample was collected. The samples were analyzed using an Automated Breath ^13^C Analyzer Isotope Ratio Mass Spectrometer (ABCA-IRMS) (SerCon-Cheshire, United Kingdom) at the Stable Isotope Center, at the Universidade Estadual de São Paulo (UNESP). The cutoff value for *H. pylori* infection to be considered present was 4‰ for the difference between the values obtained from the final and baseline breath samples

The data were analyzed using the R statistical software, version 4.0.3 (public domain). The significance level was taken to be P < 0.05.

This study was approved by the local Research Ethics Committee (CAAE 25856119.7.0000.5411) on December 4, 2019.

## RESULTS

The study sample consisted of 161 asymptomatic children (80 males and 81 females). The age range was 5-13 years, and the mean age was 7.8 ± 1.7 years. *H. pylori* infection was identified in 33/161 children (20.5%), i.e. 15/80 females (18.7%) and 18/81 males (22.2%). Half of the children were male and 45.3% were between 5 and 7 years of age **(Table 1)**. The results regarding sociodemographic, economic and sanitary condition data did not differ between children with and without *H. pylori* infection (Table 1).

**Table 1:** Sociodemographic characteristics of the study sample in relation to presence or absence of *H. pylori* (Hp) infection

Characteristic		Hp-positive n = 33 (%)	Hp-negative n = 128 (%)	Total n = 161 (%)	P value
**Gender**					
	Male	18 (54.5)	63 (49.2)	81 (50.3)	0.697^a^
	Female	15 (45.5)	65 (50.8)	80 (49.7)	
**Age (years)**					
	5 to 7	17 (51.5)	56 (43.8)	73 (45.3)	
	8 and 9	8 (24.2)	48 (37.5)	56 (34.8)	0.355^b^
	10 to 13	8 (24.2)	24 (18.7)	32 (19.9)	
**First born**					
	Yes	11 (33.3)	42 (33.6)	53 (33.5)	0.977^b^
	No	22 (66.7)	83 (66.4)	105 (66.5)	
**Siblings**					
	≤ 2	22 (68.8)	89 (71.2)	111 (70.7)	0.829^a^
	> 2	10 (31.3)	36 (28.8)	46 (29.3)	
**Bed-sharing**					
	Yes	7 (21.2)	29 (22.8)	36 (22.5)	0.842^b^
	No	26 (78.8)	98 (77.2)	124 (77.5)	
**Nº of rooms**					
	≤ 2	22 (71.0)	91 (72.2)	113 (72.0)	0.889^b^
	> 2	9 (29.0)	35 (27.8)	44 (28.0)	
**Nº of people in household**					
	> 4	22 (66.7)	79 (62.2)	101 (63.1)	0.690^a^
	≤ 4	11 (33.3)	48 (37.8)	59 (36.9)	
**Nº of children**					
	≤ 2	21 (67.7)	86 (71.7)	107 (70.9)	0.663^a^
	> 2	10 (32.3)	34 (28.3)	44 (29.1)	
**Water supply**					
	Treated piped water	33 (100)	123 (96.9)	156 (97.5)	0.302^b^
	Underground well	0 (0)	4 (3.1)	4 (2.5)	
**Family education**					
	None	1 (3.0)	0 (0)	1 (0.6)	
	Elementary	3 (9.1)	19 (15.0)	22 (13.8)	0.585^b^
	High school	22 (66.7)	86 (67.7)	108 (67.5)	
	College/university	7 (21.2)	22 (17.3)	29 (18.1)	
**Family income (minimum monthly wages)** *					
	≤ 1	7 (23.3)	24 (19.8)	31 (20.5)	
	1-2	11 (36.7)	58 (47.9)	69 (45.7)	0.497^b^
	2-3	9 (30.0)	23 (19.0)	32 (21.2)	
	> 3	3 (10.0)	16 (13.2)	19 (12.6)	

* 1 minimum monthly wage = R$ 1,048.00 (approximate U$ 260.00) in February 2020;

a from Fisher's exact test;

b from Pearson's chi-square test.

30.4% of the children had previous records of upper gastrointestinal symptoms, which consisted of *H. pylori* infection in only 26.5% of these cases. The frequency of prior gastrointestinal symptoms was 39.4% among children with *H. pylori* infection and 28.1% in the non-infected group (P > 0.05).

A family history of gastritis was reported in relation to 50% of the children with *H. pylori* infection and 48.2% of the non-infected children (P > 0.05). Presence of a peptic ulcer was reported in relation to 32.3% of the children with *H. pylori* infection and 20.2% in the non-infected group (P > 0.05).

## DISCUSSION

In this study, we found that the *H. pylori* infection rate was 20.5% among 161 asymptomatic school children living in a single community. This was significantly lower than the *H. pylori* infection rate of 52% previously recorded among symptomatic children living in the same region, despite the differences in age and diagnostic methods between the groups of symptomatic and asymptomatic children.^6,7^

A recent systematic review and meta-analysis on the prevalence of *H. pylori* infection in Latin America and the Caribbean reported an *H. pylori* infection rate of 48.3% among children and adolescents.^8^ Similar rates have been registered in this age group in Brazilian studies conducted in São Paulo.^9,10^ Prevalence rates higher than 40% have been reported among asymptomatic children up to six years of age in the northeastern region.^11,12^ In these studies, low socioeconomic status and poor hygiene conditions were the main risk factors for *H. pylori* infection.

The 20.5% rate of *H. pylori* infection identified in the present study is similar to the values recorded in developed countries, such as 22.1% in Denmark, 24.6% in Australia and 25.4% in the United States.^1,2,13^ Our result is lower than the 32.6% worldwide prevalence of *H. pylori* infection among children, reported by Zamani et al. According to these authors, the rate of *H. pylori* infection can be considered to be an additional benchmark for the socioeconomic and health status of a region or a country.^2^

In the present study, only 14.3% of all the children and 12.1% of the children with *H. pylori* infection had parents whose educational level was low. Thus, the educational background of most of our study population was good. Moreover, we did not find differences in family income, number of people in the household, number of children or occurrence of bed-sharing, between children with and without *H. pylori* infection. The similarity between the two groups of children may be explained by particular conditions of the city of Botucatu, which is located in the most developed region of Brazil and has one of the highest human development indexes (HDIs) of the country (0.8), similar to the HDI reference values of developed countries.^14,15^ These findings document the adequate socioeconomic and hygiene conditions of the study population and highlight the relevance of socioeconomic status and family education in relation to protection against *H. pylori* infection.^2,16^

In this study, previous reports of gastrointestinal symptoms were observed in relation to 30.4% of the children, which consisted of *H. pylori* infection in only 26.5% of these cases. Thus, the majority of the children with previous records of gastrointestinal symptoms did not have *H. pylori* infection. These findings are in agreement with previous studies, which did not find any relationship between gastrointestinal symptoms and *H. pylori* infection.^7,17^

A family history of gastritis was reported in half of the children with *H. pylori* infection, and peptic ulcer disease in 32.3% of the children with *H. pylori*. These findings highlight the importance of transmission from one human to another, as the main route for acquisition and transmission of *H. pylori* infection.^18^

## CONCLUSION

The prevalence of *H. pylori* infection among asymptomatic children in southeastern Brazil is lower than the prevalence recorded among symptomatic children living in the same region and similar to the rates of *H. pylori* infection observed in developed countries.
